# Recovery from post-traumatic stress disorder after a flood in China: a 13-year follow-up and its prediction by degree of collective action

**DOI:** 10.1186/s12889-015-2009-6

**Published:** 2015-07-07

**Authors:** Shimin Hu, Hongzhuan Tan, Reuben Cofie, Jia Zhou, Tubao Yang, Xuemin Tang, Aizhong Liu

**Affiliations:** Department of Epidemiology and Health Statistics, School of Public Health of Central South University, 110 Xiangya Road, Changsha, Hunan 410007 PR China

**Keywords:** Floods, Post-traumatic stress disorder, Chronicity, Prognosis factors, Epidemiology

## Abstract

**Background:**

Victims exposed to serious traumatic experiences may develop post-traumatic stress disorder (PTSD) and suffer this mental health problem for a long time. Different types of trauma displayed a chronicity rate of PTSD within the range of 6.3-68.9 %. As one of the most common and severe natural disasters, the natural progression of flood related PTSD has not been revealed. The aim of this study was to estimate the chronicity rate and identify the prognostic factors of PTSD in flood victims.

**Methods:**

Flood victims, who were over the age of 16 and diagnosed with PTSD in 2000 in Huarong, Ziyang, and Anxiang counties of Hunan province, China, were enrolled in this survey. Current probable PTSD was analyzed using the PTSD Checklist-Civilian version. Data were collected in face-to-face interviews and analyzed using univariate analysis and multiple logistic regression models.

**Results:**

The rate of current probable PTSD was 15.4 %. The current occurrences of re-experiencing, avoidance/numbing, and hyper-arousal symptom groups were 69.3 %, 17.2 %, and 50.2 %, respectively. Significant prognostic factors for current probable PTSD were flood-related stressors (e.g., life-threatening experiences, extreme physical adversity, and extreme psychological adversity) and frequency of general collective action. The relationships still existed when taking the fluctuation of frequency of general collective action into consideration. Gender and education level showed no influence on the recovery from PTSD. The impact of age in this study was inconsistent; in the 2000 model, around 2006 model, around 2013 model, and all FGCA model, older age was positive prognosis factor for PTSD; in the univariate analysis and fluctuation model, age showed no influence on the recovery from PTSD.

**Conclusions:**

Our findings indicated that PTSD can persist 13 years after a flood. Demographic characteristics (e.g., age, gender, and education level) seem to have no influence on the recovery from PTSD. Trauma-related stressors and social participation are important predictors for remission from chronic PTSD.

## Background

Post-traumatic stress disorder (PTSD) is a severe and complex disorder precipitated by exposure to psychologically distressing events and is characterized by persistent intrusive memories about the traumatic event, persistent avoidance of stimuli associated with the trauma, and persistent symptoms of increased arousal [1].

A systematic review which comprised about 10,500 participants with PTSD demonstrated that between 18 % and 50 % of patients experienced a stable recovery within 3-7 years; the remaining subjects either facing a recurrent or a more chronic course [2]. However, the development of chronic PTSD varies due to different types of trauma. For example, follow-up studies in subjects with PTSD after oil platform disasters [3, 4], World Trade Center Disaster [5], Oklahoma city bombing [6], Buffalo Creek dam collapse [7], and war [8] displayed a chronicity rate of PTSD within the range of 6.3-68.9 %. Chronic PTSD can persist for a long time. For instance, a previous study revealed that chronic PTSD can persist for over 27 years [3]. A long disease course leads to huge burdens on society, patients’ families, and patients themselves. Importantly, patients with chronic PTSD experience significantly impaired quality of life. Study of Doctor et al demonstrated that chronic PTSD patients are willing to give up 34 % of their life span to live the rest of their life without PTSD [9]. However, only a few studies have focused on the long-term natural development of PTSD. In addition, there is a lack of studies currently on the chronicity of PTSD in victims that survived natural disasters, such as flood.

Flood is one of the most common and severe natural disasters [10, 11]. No study to our knowledge has focused on the prognosis of PTSD for more than 10 years after a flood. Many areas in China frequently experience floods. A severe flood hit China’s Hunan province in 1998 and 1999, leaving hundreds of thousands of residents homeless. Our previous studies focused on flooding hazards and risk factors of PTSD, and revealed that the prevalence of PTSD in these survivors was 9.2 % one year after the flood [12–14]. The natural progression of PTSD in these victims remains unknown.

In this study, we analyzed the current status of PTSD and associated prognostic factors in fraction of these victims 13 years after the flood.

## Methods

### Ethics statement

The protocol in this study was approved by the Ethics Committee of the Institute of Clinical Pharmacology, Central South University, and signed informed consent was obtained from each interviewee. No minor or child was enrolled in this study.

### Participants

This study was a subsequent follow-up study of a large community-based survey. The previous survey had covered 8 counties (Yueyang, Lingxiang, Huarong, Qianlianghu, Ziyang, Anxiang, Datonghu, and Longshan) that had been directly exposed to the 1998–1999 flood in Hunan province, China. All enrolled family members who were 16 years of age or over were asked to participate. Between January and May 2000, trained research assistants carried out face-to-face interviews (with on-site supervision by psychologists), using a pre-constructed questionnaire. The subjects were interviewed to ascertain PTSD and to collect demographic data. A total of 2336 (9.2 %) subjects were diagnosed as probable PTSD-positive individuals among a total of 25,478 study subjects according to the DSM-IV criteria [12].

The participants for the current study were enrolled from 3 counties: Huarong, Ziyang, and Anxiang, which have not had flood since 2000. In these three counties, 851 victims had been diagnosed as probable PTSD-positive individuals. All 851 victims were recruited for this follow-up study.

### Data collection

439 victims were interviewed from house to house in Huarong in December, 2012; 412 victims were interviewed from house to house in Ziyang and Anxiang from May to June, 2014. Before survey, we wrote an investigator manual with the collaboration of psychologists and trained 8 interviewers, who worked at the local Centres for Disease Control and Prevention and had a bachelor’s degree or higher. With a unified understanding of all items, these 8 interviewers carried out face-to-face interviews using a questionnaire to ascertain PTSD and to obtain characteristics of each interviewee. The interviewers received onsite supervision from psychologists.

### PTSD outcome

The diagnosis of PTSD was made according to PTSD Checklist-Civilian version (PCL-C) questionnaire developed from DSM-IV, which was also used in our former survey in 2000 [12]. The PCL-C was highly internally consistent (α = 0.94) and had good convergent and discriminant validities [15]. The questionnaire for PTSD had been previously tested in Chinese populations and had been proven to be valid and reproducible [10]. This survey includes 17 symptoms scored as 0 = none, 1 = slight, 2 = moderate, 3 = severe, and 4 = extreme. Subjects whose score was equal to or greater than 2 were defined as positive for that symptom. The 17 symptoms of PTSD were further divided into 3 groups, representing 3 sets of diagnostic criteria: B, C, and D. Subjects were given a diagnosis of probable PTSD if Criterion B, C, and D symptoms were all positive. All suspected cases were diagnosed as ‘probable PTSD’ because the diagnosis of PTSD may not be accurate although the interviewers received on-site supervision from psychologists.

### Individual characteristics

The demographic characteristics, post-flood severe stressors, frequency of general collective action around 2006, and frequency of general collective action around 2013 were obtained during the interview. Demographic variables included gender, age, and education level. Post-flood severe stressors were measured by the question: “After 2000, have you experienced or witnessed an incident, which almost caused death or serious injury and caused you to be frightened?” Respondents could answer either “yes” or “no”. The question about post-flood severe stressors was put right after questions about demographic characteristics. If the answer of post-flood severe stressors was ‘yes’, the interview would be wrapped up.

Questions about “frequency of general collective action” were put together and shared a description: “General collective action means participating in activities organized by the following groups: political party, trade union, environmental group, parents’/school association, tenants’/residents’ group or neighborhood watch, church organization, voluntary service group, pensioners group/organization, social club/working men’s club, sports club, and the Women’s Institute [16].” Frequency of general collective action around 2006 was measured by the question: “Recalling the situation around 2006, to what extent did you take part in general collective action?” Frequency of general collective action around 2013 was measured by the question: “According the situation this year, to what extent do you take part in general collective action?” Respondents could answer 1 = “never”, 2 = “occasionally”, or 3 = “frequently”. Flood-related stressors and frequency of general collective action in 2000 were assessed in previous survey. Frequency of general collective action in 2000 was measured by the question: ‘To what extent did you take part in general collective action?’ Respondents could answer 1 = “never”, 2 = “occasionally”, or 3 = “frequently”. Flood-related stressors include: life-threatening experiences (e.g., narrow escape from flood waters requiring emergency rescue), death of a loved one, victimization after the flood (e.g., robbery–assault), physical illness/injury caused or exacerbated by the flood, extreme physical adversity (e.g., difficulty obtaining food or clothing), extreme psychological adversity (e.g., living in circumstances where the respondent had to use the toilet or change clothes without privacy), major property loss, income loss, and housing adversity (e.g., multiple moves). Victims answered each question with either a “yes” or “no”.

### Statistical methods

Individual factors between interviewed victims and unreachable victims were first compared by *χ*^2^ test. Although the PCL-C had been used widely to diagnose PTSD, nine items on the questionnaire have no word about “stressor” in the stems. In order to avoiding the influence from other flood unrelated stressors, we excluded the victims who have experienced post-flood severe stressors in all following analyses.

Current probable PTSD positive rates for two interviewed time (2012 and 2014) in Huarong, Ziyang and Anxiang counties were described respectively and compared by χ^2^test, and then the date was put together and the positive case numbers and rates of each symptom cluter were described. To correct for possible confounding, the effect of the three counties was taken into consideration by including two dummy variables as predictors into the model.

In order to reveal the impact of general collective action on PTSD, a new indicators, fluctuation of frequency, was created. Score of 2006 minus score of 2000 is result1; score of 2013 minus score of 2006 is result2. If result1 and result2 are both 0, the result of “the fluctuation of frequency” is “no change”. If result1 and result2 are both positive number, or one of them is positive number, the other is 0, the result of “the fluctuation of frequency” is “becoming more frequent”. If result1 and result2 are both negative number, or one of them is negative number, the other is 0, the result of “the fluctuation of frequency” is “becoming less frequent”. If one of result1 and result2 is negative number, the other is positive number, the result of “the fluctuation of frequency” is “fluctuating”.

The rates of current probable PTSD among victims of different gender, age, education level, flood-related stressors, frequency of general collective action in 2000, around 2006, and around 2013, the fluctuation of frequency were compared respectively by univariate logistic regression models. Adjusted ORs for current probable PTSD were estimated with multiple logistic regression models. A total of 5 models were analyzed: frequency of general collective action in 2000, around 2006, around 2013, the fluctuation of frequency were included into 4 different models as independent variable, respectively; in “All FGCA model”, frequency of general collective action in 2000, around 2006, around 2013 and the fluctuation of frequency were all included. Gender, age, education level, and flood-related stressors were included as independent variables in every model. All analyses were performed with SPSS Version 18.0.

## Results

### Baseline data

851 individuals were selected for this study. Of the 851 selected study subjects, 284 were visited at their home, 57 died of old age, 121 migrated to other villages or counties, and 389 temporarily left to find work in other cities. The availability for follow-up is only 33.4 % (284/851). Table 1 displays a comparison of baseline information collected in 2000 between the interviewed and unreachable victims. The results indicate that the distribution of gender, educational level, flood-related stressors, and frequency of general collective action are similar between interviewed and unreachable victims. There were more young subjects among the unreachable victims.

**Table 1:** Comparison of Individual characteristics between subjects interviewed (n = 284) and unreachable (n = 567)

Individual characteristics	Interviewed		Unreachable		*χ* ^2^	P
	Number	%	Number	%		
Gender						
Male	153	53.9	278	49.0	1.776	0. 183
Female	131	46.1	289	51.0		
Age						
29-57	174	61.3	423	74.6	16.071	0.000
58-81	110	38.7	144	25.4		
Education Level						
Elementary school or below	131	46.1	231	40.7	2.246	0.134
Middle school or above	153	53.9	336	59.3		
Flood-related stressors						
0	118	41.5	278	49.0	4.384	0.112
1	69	24.3	125	22.0		
≥2	97	34.2	164	28.9		
Frequency of General Collective Action in 2000						
Never	62	21.8	152	26.8	2.491	0.288
Occasionally	118	41.5	220	38.8		
Actively	104	36.6	195	34.4		

Among the 284 interviewed subjects with PTSD, 17 subjects were excluded from the follow up analysis because they experienced post-flood severe stressors. Ultimately, 284 questionnaires were distributed and 267 valid questionnaires were received. Of these 267 victims, the average age is 53.55 years (Standard Deviation = 10.60), and per capita annual income is 6903.48 yuan (Standard Deviation = 5540.70). According to the National Bureau of Statistics of China, per capita annual income of the nation’s rural households is 7916.6 yuan in 2012.

### PTSD symptoms

Descriptive statistics of the positive rates of probable PTSD symptoms of interviewed subjects are presented in Table 2. Current probable PTSD positive rate of Huarong and Ziyang + Anxiang were 14.6 % and 16.3 % (*χ*^2^ = 0.144, P = 0.705, not shown in table). The prevalence rate of current probable PTSD of total victims was 15.4 %. The re-experiencing cluster (B) of symptoms was 69.3 % positive, avoidance/numbing cluster (C) was 17.2 % positive, and hyper-arousal (D) was 50.2 % positive. The positive rate of Group C was much lower than group B and D. Although the dates were not indicated in the table, the positive rates of 3 symptom clusters were undoubtedly all 100 % in 2000. Group C had biggest drops in positive rate (P < 0.000, by *χ*^2^ test, not shown in table).

**Table 2:** DSM-IV criterion symptoms of PTSD reported by 267 subjects with PTSD 13-year after first exposure to flood

	N of subjects	N of positive Number	Percent
PTSD, Huarong	144	21	14.6
PTSD, Ziyang and Anxiang	123	20	16.3
PTSD, total interviewed victims	267	41	15.4
B. Re-experiencing	267	185	69.3
C. Avoidance/Numbing	267	46	17.2
D. Hyper-arousal	267	134	50.2

### Relationship between individual factors and chronic PTSD

The risk of chronic PTSD increased in victims who had experienced more flood-related stressors and lower frequency of general collective action. Although frequency of general collective action could change over 13 years, the data of 2000, around 2006, around 2013 revealed a similar trend: subjects who had higher frequency of general collective action had better prognosis of PTSD. Because of the limited sample size, fluctuation results had no statistical significance, but showed the same trend. In the “all FGCA model”, frequency of general collective action around 2006 was a significant predictor of the chronicity of PTSD. In both univariate and multivariate analyses, gender and education level showed no influence on the recovery from PTSD. However, the results of age were different. In the 2000 model, around 2006 model, around 2013 model, and all FGCA model, older age was positive prognosis factor for PTSD (Table 3).

**Table 3:** Independent prognosis factors for current PTSD, expressed in OR and adjusted ORs of 5 models

Individual characteristics	Univariate analysis			2000 model	Around 2006 model	Around 2013 model	Fluctuation model	All FGCA model
	Number	Prevalence	OR	Adjusted OR	Adjusted OR	Adjusted OR	Adjusted OR	Adjusted OR
Counties								
Huarong	144	14.6	Reference					
Ziyang	46	30.4	2.562*	1.779	1.443	1.704	3.138*	1.069
Anxiang	77	7.8	0.495	0.504	0.437	0.597	0.538	0.324
Gender								
Male	141	13.5	Reference					
Female	126	17.5	1.358	1.085	1.108	1.132	1.387	1.698
Age								
29-57	159	18.9	Reference					
58-81	108	10.2	0.488	0.375*	0.284*	0.366*	0.499	0.323*
Education Level								
Elementary school or below	123	13.8	Reference					
Middle school or above	144	16.7	1.247	0.808	0.831	0.747	0.749	1.122
Flood-related stressors								
0	105	3.8	Reference					
1	66	19.7	6.196**	7.235**	8.876**	6.785**	5.917**	11.733**
≥2	96	25.0	8.417***	9.645***	12.341***	9.634**	9.036***	17.512***
FGCA in 2000								
Never	61	32.8	Reference					
Occasionally	113	13.3	0.314**	0.268**				1.255
Actively	93	6.5	0.141***	0.133***				0.218
FGCA around 2006								
Never	54	50.0	Reference					
Occasionally	116	8.6	0.094***		0.065***			0.021***
Actively	97	4.1	0.043***		0.031***			0.076*
FGCA around 2013								
Never	55	45.5	Reference					
Occasionally	103	11.7	0.158**			0.182***		3.629
Actively	109	3.7	0.046***			0.045***		0.906
Fluctuation of FGCA								
No change	152	15.8	Reference					
Getting frequent	61	6.6	0.374				0.320	0.677
Getting less frequent	38	28.9	2.173				2.003	6.893
Fluctuation	16	12.5	0.762				0.846	0.345

*FGCA* Frequency of General Collective Action; *p < .05; **p < .01; ***p < .001

## Discussion

The chronicity of PTSD in victims of flood has not been well addressed. This study investigated the chronicity rate of PTSD and associated prognostic factors in victims of flood 13 years after the traumatic experience. This is the first follow-up study that examined flood victims beyond 10 years after the event. This study revealed that the chronicity rate of PTSD was 15.4 %, and the current occurrences of B (Re-experiencing), C (Avoidance/Numbing) and D (Hyper-arousal) symptom clusters were 69.3 %, 17.2 % and 50.2 %, respectively. Flood-related stressors and frequency of general collective action have been found to be associated with recovery from PTSD. This study provided an insight on the prognosis of flood-related PTSD.

The prognosis of PTSD in flood victims had been previously studied in America (16 months, 72.7 %) and Mexico (24 months, 41.7 %) with a short follow-up term [17, 18]. In our study, the chronicity rate of PTSD was 15.4 % in victims 13 years after a flood, which is lower than reported in previous studies with shorter follow-up periods [17, 18]. Compared to previous studies with similar follow-up periods, the chronicity rate of PTSD found in our study is lower than chronicity rates found among oil platform disaster victims (10 years, 28.8 %; 27 years, 27.5 %) [3, 4], Oklahoma City bombing survivors (7 years, 65.9 %) [6], Buffalo Creek dam collapse victims (14 years, 63.6 %) [7] and war veterans (14 years, 44.9 %) [8], but higher than the chronicity rate found among firefighters involved in World Trade Center Disaster (9 years, 12.3 %) [5]. These varying results suggest that the nature and severity of different types of disasters and differences in populations may be responsible for the differences in the chronicity of PTSD. But all of these studies revealed that long-term chronicity of PTSD exists.

Our survey revealed that the rate of group B, C, and D syndromes after 13 years of progression was 69.3 %, 17.2 % and 50.2 %, respectively. The positive rate of group C symptoms was similar to the positive rate of PTSD. Several retrospective studies have suggested that Group C criteria are a marker for PTSD [17, 19, 20]. Previous studies demonstrated that a consistently large majority (75 % to 100 %) of individuals meeting symptom group C meet full PTSD criteria [17, 19]. Pietrzak et al suggested that PTSD symptoms comprising criterion C largely account for associated psychopathology and functional difficulties in individuals exposed to trauma [21]. The study of Whitman et al demonstrated that the C group not only had a lower positive rate but also had a slower onset in the first month after trauma exposure [22]. However, this phenomenon was not observed in other studies [20, 23, 24]. In 2000, positive rate of group B, C, and D syndromes were all 100 %. In 2013, symptom group C had a better recovery situation over the past 13 years. These findings suggest that remission of symptoms of the avoidance/numbing symptom cluster of PTSD appears to be pivotal for recovery from PTSD [20, 25].

This study demonstrated that victims who experienced more flood-related stressors experienced worse recovery from PTSD. This is in agreement with most of previous studies [3, 5, 7, 8, 26, 27], but in disagreement with a few studies [6]. Flood-related stressors in our study included some factors reflecting the quality and privacy of life during post-disaster temporary resettlement. These may suggest that keeping victims safe with adequate food and clothing is very important but not enough post disaster. The government should take the privacy of victims’ post-traumatic living conditions into consideration. An important finding in this study is that victims who participated in general collective action more frequently experienced better recovery from PTSD. Koenen et al found that, among Vietnam veterans with PTSD, those who engaged in more community activities are more likely to show remission from PTSD [8]. Participating in general collective action can strengthen victims’ contact with society and may have a positive impact on prognosis of PTSD through individual appraisal processes (i.e. primary and secondary appraisal), social support, and coping behavior [28]. Our study therefore suggests that more social participation is beneficial to the recovery from PTSD. The results of age in this study were inconsistent; some of the results suggested older age at the time of exposure to the flood was a positive prognosis factor for PTSD. This inference needs an in-depth study to confirm.

We acknowledge that there are some limitations to this study. First, the follow-up rate, although low, is typical of longitudinal studies of nature disasters [7, 27]. However, the distribution of gender, education level, flood-related stressors and frequency of general collective action in 2000 is similar between interviewed and unreachable victims. This suggests that the analysis related to stressors and collective action is reliable. Although our sample size is small, the results of our study can make some contribution to the prevention of chronic PTSD and at least, arousing attention about chronic PTSD. Second, PTSD symptoms often fluctuate. Observations on relapsing-remitting and late-onset symptoms’ trajectories may add more information to the current one level measurement. Indeed, observation of the fluctuating changes in symptoms is crucial to PTSD management. However, the long-term chronicity rate may be more important for a social study and prognostic factor analysis. Third, because a dozen years had passed, the situation of some individual factors, to some extent, may change. We measured the exact situation in 2000 and around 2013, but had to estimate the situation in between these years. Fourth, the OR we got may be overstated because of the possible mutual influence of PTSD and FGCA.

## Conclusion

This study suggests the existence of chronicity in PTSD victims of flood. Remission of the avoidance/numbing symptoms plays an important role in the recovery from PTSD. More social participation seems to be beneficial to the recovery from PTSD, and re-building a high quality life after a disaster with help from society or government could reduce the development of chronic PTSD in flood victims.
